# Etiology, Clinical Profiles, and Outcomes of Acute Encephalitis Syndrome Cases Admitted to a Tertiary Care Center in Myanmar in 2023

**DOI:** 10.3390/diagnostics14192248

**Published:** 2024-10-09

**Authors:** Aung Kyaw Kyaw, Ohnmar, Zin Nwe Win, Sai Kyaw Win, Zarni Myint Shwe, Kyaw Lwin Show, Nan Aye Thida Oo, Mya Thandar Win, Khin Zarchi Aung, Win Pa Pa Naing, Phyu Phyu Lay, Hlaing Myat Thu, Zaw Than Htun

**Affiliations:** 1 Department of Medical Research, Ministry of Health, Yangon 11191, Myanmar; 2 Department of Neurology, University of Medicine 1, Yangon General Hospital, Ministry of Health, Yangon 11111, Myanmar

**Keywords:** acute encephalitis syndrome, infectious cause, autoimmune encephalitis, adult, Myanmar, 2023

## Abstract

Background/Objectives: The diagnosis of encephalitis is a challenging problem due to the heterogeneity of clinical presentations. The objective was to determine the etiology, clinical features, laboratory parameters, radiological findings, and in-hospital outcome of acute encephalitis syndrome (AES) cases in Myanmar. Methods: A prospective descriptive study was conducted at the Neuromedical Ward of Yangon General Hospital from March to August 2023. Eighty-one AES cases were enrolled, and cerebrospinal fluid (CSF) samples were collected. A Qiastat ME Panel was used to detect viral, bacterial, and fungal pathogens. Results: Seventeen out of eighty-one (21%) cases were non-encephalitis with alternative definite diagnosis. Among the remaining 64 encephalitis cases, the exact infectious and immune etiologies were identified in 31 of 64 cases (48.4%); 26 of these (83.9%) were due to infectious causes and 5 (16.1%) were immune encephalitis. Among the infectious causes, six Herpes Simplex Virus-1-, one bacteriologically confirmed and seven probable *Mycobacterium tuberculosis*-, three *Haemophilus influenzae*-, two *Streptococcus pneumoniae*-, one *Streptococcus pyogenes*-, one Varicella-Zoster Virus (Ramsay Hunt Syndrome with meningoencephalitis)-, and two *Cryptococcus neoformans*-infected patients and rare causes such as *Listeria monocytogenes*, *Burkholdelria cepacia*, *Sphingomonas paucimobilis*, and *Aspergillus* were identified. One case was a dual infection with *Haemophilus influenzae and Cryptococcus neformans*. Abnormal protein levels and CSF pleocytosis were significantly higher among bacterial causes (*p* < 0.05). In total, 6.45% (2/31) of encephalitis patients with identified causes and 12.12% (4/33) of those without an identified organism had poor outcome. Conclusions: Herpes encephalitis and tuberculous meningoencepalitis were the commonest. This study highlighted that molecular testing with a multidisciplinary approach is required to ensure the right treatment on time.

## 1. Introduction

Encephalitis is a complex clinical problem due to a myriad of etiologies and pathogeneses with high mortality rate. It is often complicated by prolonged and permanent neurologic deficits. Encephalitis can be caused not only by infections but also by immune-mediated mechanisms. The incidence of the disease varies depending on the economic status of the country, but it is generally between 3.5 and 7.4 per 100,000 patients per year [1]. While the incidence in the population is not high, the morbidity and mortality rates are significant, making it a persistent public health problem worldwide. The mortality rate of encephalitis cases ranges from 3.8% to 7.4% [2].

The etiologies of encephalitis are mainly classified into two groups: infectious and immune-mediated causes. Although infectious causes are the most common, the reported number of autoimmune encephalitis cases has increased in both adults and pediatric patients due to advancements in diagnostic facilities and treatment options [3]. There are various types of autoimmune encephalitis; in young adults, particularly women, anti-N-methyl-D-aspartate receptor antibody (anti-NMDAR Ab) encephalitis is common, while in late adulthood, anti–leucine-rich glioma-inactivated 1 (anti-LGI1) encephalitis is the most prevalent [4]. Prompt diagnosis is essential in cases of autoimmune encephalitis for the initiation of effective and appropriate treatment [5].

Among the infectious group, viral causes are the most common, and both Deoxyribonucleic Acid (DNA) and Ribonucleic acid (RNA) viruses can present with encephalitis [6]. The herpes virus group, Varicella-Zoster Virus (VZV), Japanese Encephalitis (JE), Zika (ZKV), Dengue (DENV), Chikungunya (CHIKV), Influenza, Adeno, and Human Immunodeficiency (HIV) viruses are common viruses that can cause encephalitis. Some bacterial infections including *Haemophilus influenzae*, *Escherichia coli*, *Streptococcus pneumoniae*, *Streptococcus pyogenes*, *Neisseria meningitidis*, *Mycobacterium tuberculosis*, *Mycoplasma pneumoniae*, *Listeria monocytogenes*, *Leptospira*, and *Salmonella typhi*, can also present as encephalitis. *Cryptococcus neoformans* and *Aspergillus* should also be considered in the etiology of encephalitis. The common causative microorganisms can vary with the seasons, geographical regions, and the immunity of the patients [7].

Even in developed countries, diagnostic and management guidelines are based on the prevalence of the diseases causing encephalitis. Genetic background, immunization status, and geographical location are essential factors to consider when developing diagnostic and management guidelines, such as adding ampicillin to empiric antibiotic therapy in areas with a high prevalence of *Listeria monocytogenes*, or conducting arboviral screening tests in tropical countries.

There are still limited data on the prevalence of viral, bacterial, fugal, and immune-mediated encephalitis and meningoencephalitis cases among the adult population in Myanmar. Data obtained from this research are valuable as baseline information for the surveillance of vaccine-preventable diseases, help close the gap in disease surveillance for emerging infectious diseases causing encephalitis, and support considerations for routine vaccination and other preventive measures. Furthermore, in Myanmar, there is limited information regarding the common organisms causing encephalitis in adult patients and the proportion of both infectious and immune-mediated causes of acute encephalitis cases in the adult population. To prioritize laboratory tests, surveillance was conducted to identify the etiology, clinical profiles, laboratory investigations, and outcomes of encephalitis patients admitted at the Neuromedical Ward at Yangon General Hospital during 2023.

## 2. Materials and Methods

A prospective descriptive study was conducted at a tertiary care center, the Neuromedical Ward of Yangon General Hospital (YGH), from March to August 2023. This study was approved by the Institutional Review Board of the Department of Medical Research with the approval number Ethics/DMR/2022/20. Surrogate written informed consent was obtained before enrolling patients into the study.

### 2.1. Patients’ Recruitment

All patients presenting with acute encephalitis syndrome (AES) and admitted to the Neuromedical Ward during the study period were recruited. AES is defined as a patient with an acute onset of fever (>38 °C) within the preceding 7 days, accompanied by one or more of the following clinical features: altered mental status or seizure [6]. Patients with previously diagnosed tuberculosis (any site) and malignancy, cirrhosis of liver, end-stage renal disease, stroke, or epilepsy cases were excluded from the study.

### 2.2. Study Procedures

Demographic profiles, clinical presentations, outcome at discharge, immunocompromised status, electroencephalogram (EEG) results, neuroimaging (Computed Tomography (CT)/Magnetic Resonance Imaging (MRI)) findings were recorded. The final diagnosis of the cause of encephalitis was made by two neurologists (one junior and one senior) from the study hospital. Cerebrospinal fluid (CSF) samples were analyzed for molecular tests, as well as biochemical and microscopic investigations. If the results were negative for infectious diseases, anti-NMDAR antibodies were tested.

### 2.3. Laboratory Tests

In total, eight bacterial pathogens (Neisseria meningitis, *Haemophilus inflenzae*, *Streptococcus pneumoniae*, *Streptococcus agalactiae*, *Sterptococcu pyogens*, *Listeria moncytogenes*, *Escherichia coli*, and *Mycoplasma pneumoniae*), six viral pathogens (Herpes simplex virus 1 (HSV-1), Herpes Simplex Virus (HSV-2), Human Herpes Virus 6 (HHV-6), Entero virus Human Parechovirus, and Varicella Zoster Virus (VZV)), and one fungal pathogen (Cryptococcus neoformans) were checked on CSF specimens by Real-time PCR-based QIAstat-Dx ME Panel (Qiagen, Hilden, Germany) using QIAstat-Dx Analyzer 1.0. To identify four arboviruses (Japanese Encephalitis, West Nile, Dengue, and Chikungunya Viruses), viral RNA was extracted from CSF samples using Qiagen Mini Viral RNA extraction kits (Qiagen, Hilden, Germany), according to the manufacturer’s instruction. Conventional one-step Reverse Transcription PCR (Takara one step RT-PCR, Takara, Osaka, Japan) was carried out to detect JEV, DENV, ZIKV, and CHIKV genomes using specific primers. All the experiments were conducted according to the procedures described in previous studies [8,9,10]. The molecular tests were performed at the Pathology Research Division, Department of Medical Research, Yangon.

In this study, laboratory-confirmed, probable, and possible diseases were defined according to the criteria for the diagnosis of acute encephalitis by an infectious agent [6]. For the diagnosis of autoimmune encephalitis, a cell-based indirect immunofluorescence (EUROIMMUNE, Lubeck, Germany) assay for anti-NMDAR Ab was measured from either serum or CSF samples [11]. Laboratory-confirmed infection was operationally defined as either the nucleic acid detection of microorganisms or the isolation of pathogens from culture or other routine diagnostic tests. Probable TB cases were operationally defined based on clinical features and response to anti-TB treatment [6].

### 2.4. Statistical Analysis

Data entry was carried out using Microsoft Excel and analysis was performed using STATA Software, Version 15 (STATA Corp., College Station, TX, USA). Descriptive statistics for demographic features, clinical findings, CSF analysis, and radiological features were presented as frequencies and percentages. The classification of causes of acute encephalitis was presented using proportions and 95% confidence intervals. Differences in demographic profiles, clinical presentations, laboratory parameters, and neuroimaging results were compared by the classification of causes of acute encephalitis using a chi-square test. Univariable analyses were applied to identify the factors associated with the survival of patients with encephalitis. A *p*-value of <0.05 was considered statistically significant.

## 3. Results

**Figure 1:**
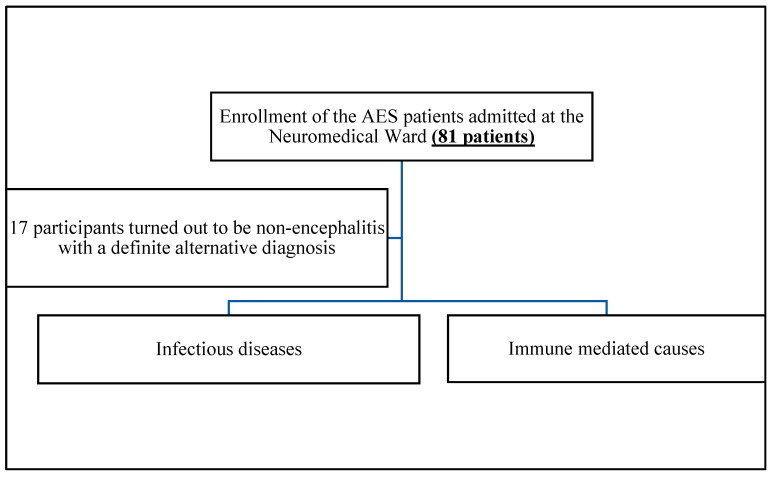
Flow chart of the study.

In this study, a total of 81 AES cases were enrolled, with 40/81 (49.4%) being males and 41/81 (50.6%) being females. Among the 81 cases, 29 patients (35.8%) were under 35 years old, 32 patients (39.5%) were between 35 and 59 years old, and 20 cases (20.7%) were over 60 years old. Of the 81 total patients, 17 cases were diagnosed as non-encephalitis with a definite alternative diagnosis such as metabolic encephalopathy, septic encephalopathy, cerebral malaria, stroke, epilepsy, or brain metastasis (Figure 1).

Out of the remaining 64 encephalitis cases, viral encephalitis cases constituted 32, pyogenic meningoencephalitis 16, tuberculous meningoencephalitis 8, immune encephalitis 6, and fungal infection 3. Six patients died among the sxity-four, and the mortality rate of encephalitis in this study was 9.37%, (95% CI, 3.3, 15.8). Among these 64 encephalitis cases, only 31/64 (48.44%) cases had exact etiological diagnosis. Of the 31 cases with an identified etiology, infectious encephalitis accounted for 83.87% (26/31), and immune causes accounted for 16.13% (5/31). Of the latter, four were anti-NMDAR ab autoimmune encephalitis and one was systemic lupus erythematosus (SLE) cerebritis. The proportion of etiology-undiagnosed encephalitis cases was 33/64 (51.56%).

The identified bacterial causes were more frequent than the identified viral causes in this study. Herpes simplex encephalitis (HSE) was the most common cause among laboratory-confirmed cases. Regarding tuberculous meningitis, only one bacteriologically confirmed case was identified by nucleic acid amplification test, and seven cases were probable tuberculous meningitis (Table 1). A case of VZV infection (Ramsay Hunt syndrome) with meningoencephalitis was detected in this study. The organisms detected in this study are shown in Table 1.

Among the etiology-identified encephalitis cases, seven were immunocompromised patients. Three probable tuberculous meningitis patients had retroviral infection, and one had end-stage renal disease (ESRD) due to prolonged diabetes mellitus. In this study, a case of plasmacytoma with post-autologous stem cell transplant on immunosuppressants, presented with acute meningoencephalitis and *Listeria monocytogenes* was detected. Interestingly, one dual-pathogen infection case was immunocompetent. The causes of encephalitis cases among immunocompetent and immunosuppressed patients are shown in Table 2.

Demographic profiles, clinical presentations, laboratory parameters, and neuroimaging results of etiologically identified encephalitis cases were compared among bacterial, viral, fungal, and autoimmune causes, as described in Table 3. Males were more affected than females in bacterial causes, while females were more affected in viral causes (*p* < 0.05). CSF pleocytosis and abnormal CSF protein levels were significantly higher in bacterial causes (*p* < 0.05) (Table 3).

Among the neuroimaging results of thirty-one etiologically identified encephalitis cases, five cases (two HSE, one tuberculous, one anti-NMDAR encephalitis, and one SLE cerebritis) showed encephalitis. Two tuberculous cases showed infection-related infarcts, one cryptococcal case showed meningitis, one *Aspergillous* case had venous sinus thrombosis and sinusitis, and the remaining twenty-two cases were normal. EEG was performed in 71.6% (58/81): normal in 39.7% (23/58), diffuse slowing in 24.1% (14/58), focal slowing in 17.2% (10/58), focal epileptiform discharges 13.8% (8/58), electrographic seizures/status epilepticus in 8.6% (5/58), and periodic lateralized epileptiform discharges (PLEDs) in 3.4% (2/58). Two cases of PLEDs were confirmed HSE cases.

Regarding the clinical outcomes of encephalitis, the mortality rate was 6/64, 9.4% [95%CI: 4.2, 19.7], and 2/31 (6.45%) of patients with identified causes had poor outcome. One was a herpes encephalitis patient who could not afford the cost of the treatment and the other was a plasmacytoma case (post stem cell transplant) with *Listeria* infection. On the other hand, 4/34 (11.8%) of encephalitis cases without identified etiology had poor outcome. Regression analysis was performed to predict the fatal outcomes, demographic factors, clinical presentations, and laboratory parameters (Table 4). No significant variables were found for determining fatal outcomes in this study.

## 4. Discussion

Central Nervous System (CNS) infection is the second most common condition among neuro-medical ward inpatients at YGH, with high morbidity and mortality rates [12]. In this study, the mortality rate was 9.38% (6 out of 64 encephalitis cases), reflecting a still high mortality. If the exact etiology is known, early diagnosis and effective treatments such as anti-viral drugs for herpes virus, antibiotics for bacterial causes, and antifungals for fungal causes, can be administered. Subsequently, the morbidity and mortality of the disease can be reduced. This also avoids the unnecessary use of antimicrobials [13]. Unlike other infections, CNS infections require parenteral and prolonged treatment with antivirals, antibiotics, and antifungals (at least 10–14 days) [13]. By identifying the exact organisms, antimicrobial therapy can be directed, significantly reducing costs and avoiding the use of unnecessary expensive antimicrobials, while posing a lower risk of adverse effects.

Since HSE is the most common cause identified in the literature, in places where there are no testing facilities, empirical acyclovir should be given. However, acyclovir nephropathy can occur in up to 48% [14]. In this study, HSE was also the most common cause. One case of HSE presented with stroke-like symptoms (sudden-onset left hemiparesis with extreme right gaze deviation) with fever started only on day 2, leading to the initial diagnosis of stroke. The CT scan of the head was normal. MRI (brain) was also reported as right temporal infarct. However, due to the patient’s confusion and EEG findings of PLEDs and electrographic seizures, a CSF study was performed. In the molecular test, HSV-1 was detected, completely changing the management approach and ensuring that both the doctors and the patient did not miss appropriate treatment for HSE. HSE is curable, but if untreated, it has grave consequences. According to the literature, empiric acyclovir therapy should be started at presentation, and the dose can be increased for VZV encephalitis cases. In this study, a case with typical features of Ramsay Hunt syndrome (right lower motor neuron facial palsy with right ear ache with redness in the right ear) was included due to subtle right sixth cranial nerve involvement and mild sensorial change. The CSF VZV PCR came back positive, indicating CNS extension, leading to the administration of intravenous acyclovir instead of oral acyclovir, which is usually prescribed for Ramsay Hurt syndrome [15]. Nucleic acid amplification testing from CSF specimens has greatly improved the ability to diagnose CNS infections, especially those caused by herpesviruses [16]. Thus, it is essential to establish the HSV PCR testing or ME panel testing in public sector laboratories.

Tubercular meningitis (TBM) is one of the leading causes of acute febrile encephalopathy in developing countries including Myanmar [17]. In this study, one lab-confirmed case and seven probable cases of TB meningoencephalitis were included, making TB one of the most common causes of encephalitis. Based on the fourth national tuberculosis survey in 2017–2018, the estimated adult pulmonary TB cases were 468/100,000 population [18]. Thus, TB should be considered at the first differential diagnosis of encephalitis and meningitis cases. Among our eight tuberculous meningoencephalitis patients, 50% were immunocompromised but all eight patients had good outcomes. This may be due to our AES criteria, which recruited only early cases of CNS tuberculosis, where early diagnosis and immediate treatment improves the outcome of TBM [19].

Although the sample size is small, *Sphingomonas paucimobillis* meningitis, *Listeria monocytogenes* and *Burkholderia cepacia*, and *Aspergillus* meningitis/encephalitis were identified. While *Sphingomonas paucimobillis* infection primarily occurs among immunocompromised patients, some studies reported it can also infect immunocompetent individuals [20], and even co-infection with *Listeria monocytogenes* has been noted in immunocompetent individuals [21]. *Burkholderia cepacia* is a rare cause worldwide, but is now becoming an emerging cause of bacterial meningitis [22,23].

*Burkhodelria cepacia* and *Aspergillus* were isolated from serum samples, while *Sphingomonas paucimobilis* was isolated from CSF samples of routine culture and antibiotic sensitivity assays. Two patients had a positive CSF culture for *Staphylococcus hominis and Staphylococcus epidemidis*, but their CSF biochemical and microscopic assay results, along with clinical features, were not consistent with pyogenic meningitis. These cases were therefore determined to be contamination. This shows that a routine CSF culture alone is not sufficient for diagnosing encephalitis.

During the study period, a case of cryptococcal meningoencephalitis (CM) was identified, which was unexpected since the patient was immunocompetent. Previous research has shown that CM cases in immunocompetent patients are underestimated and are not as rare as previously believed. Studies have shown that up to 43.5% of cases occur in immunocompetent young individuals and non-HIV/non-organ transplant patients [24,25,26]. Furthermore, among immunocompetent patients, 67% of those presenting with CM had auto-antibodies [27]. In this study, we also encountered two cases of *Haemophilus influenzae encephalitis*, which had not been commonly tested in Myanmar before. This case highlighted the need for clinicians to maintain a high index of suspicion, as *Haemophilus influenzae* might be one of the most common identifiable organisms causing encephalitis in Myanmar. Myanmar is endemic for many arboviruses such as DENV, JEV, ZKV, and CHIKV [9,10], but no cases were identified as causes of encephalitis in this study.

Immune encephalitis accounted for 16.13% of etiologically identified encephalitis cases (5/31) and 9.38% (6/64) of all presumed encephalitis cases. Four were anti-NMDAR antibody-positive, one was an SLE case, and one was presumed to be autoimmune encephalitis [28]. In a study conducted in Myanmar during 2017–2018, 26% of pediatric encephalitis cases were diagnosed as presumed autoimmune encephalitis [29].

In this study, only 31/64 encephalitis cases had a confirmed etiology. Even in developed countries, the proportion of undiagnosed encephalitis cases remains high [30]. However, many advanced diagnostic tests are now available, allowing both infectious and non-infectious causes to be identified, which can reduce mortality rates.

One of the limitations of the study is that, due to limited resources and sample size, only anti-NMDAR antibodies were tested, likely underestimating the actual number of autoimmune encephalitis cases. Furthermore, we were able to detect only a limited number of pathogens, despite the myriad of etiologies, and could not identify all infectious and non-infectious causes such as SARS-CoV-2 and influenza virus.

## 5. Conclusions

Herpes and tuberculous were the most common causes of encephalitis, followed by autoimmune encephalitis. The proportion of patients without an accurate diagnosis was high. The findings will help bridge the data gap in infectious disease surveillance in South East Asian countries, including Myanmar. Additionally, this research has explored some rare emerging infectious diseases that can cause encephalitis. This study will also assist in developing diagnostic guidelines and tools for the early detection of encephalitis. Given that Myanmar is a large country with diagnostic facilities concentrated in a few tertiary centers, these results will be valuable for managing encephalitis cases in areas with limited diagnostic resources. Molecular testing, routine laboratory tests, and clinical and radiological findings should all be utilized for early diagnosis. Moreover, many emerging and re-emerging pathogens are known to cause encephalitis, so annual surveillance should be conducted.

## Figures and Tables

**Table 1:** Classification and causes of acute encephalitis cases with exact etiology identified (*n* = 31).

Confirmed/Probable Cases (*n* = 31, [48.4%, 95% CI—36.2, 60.8])			
A. Infectious Causes (*n* = 26, [83.9%, 95% CI—65.3, 93.5])			
	Confirmed	Probable	Total (%)
1. Viral Causes (*n* = 7, [22.6%. 95% CI—10.7, 41.6])			
Herpes simplex Virus-1	6	-	6 (85.7)
Varicella Zoster Virus	1	-	1 (14.3)
2. Bacterial causes including *Mycobacterium tuberculosis* (*n* = 16, [51.6%, 95% CI—33.6, 69.2])			
*Haemophilus Influenzae*	2	-	2 (12.5)
*Streptococcus pneumonia*	2	-	2 (12.5)
*Streptococcus pyogenes*	1	-	1 (6.25)
*Listeria monocytogenes*	1	-	1 (6.25)
*Mycobacterium tuberculosis*	1	7	8 (50.0)
*Sphingomonas paucimobilis*	1	-	1 (6.25)
*Burkholderia cepacia*	1	-	1 (6.25)
3. Fungal infection (*n* = 2, [6.5%, 95% CI—1.5, 23.9])			
*Cryptococcus neoformans*	1	-	1 (50.0)
*Aspergillus*	1	-	1 (50.0)
4. Dual infection (*n* = 1, [3.2%, 95% CI—0.4, 21.6])			
*H. influenzae and Cryptococcus neoformans*	1	-	1 (100)
B. Immune-mediated cause (*n* = 5, [16.1%, 95% CI—6.5, 34.6])			
1.Anti-NMDAR immune encephalitis	4	-	4 (75.0)
2. SLE cerebritis	1	-	1 (25.0)
Total	24	7	31 (100)

*n* = number of cases. 95% CI = 95% Confidence Interval. Anti-NMDAR Ab = anti-N methyl-D-Aspartate Receptor Antibody. SLE = Systemic Lupus Erythematosus.

**Table 2:** Identified causes of encephalitis in immunocompetent and immunocompromised patients (*n* = 31).

Etiology	Immunocompetent	Immunocompromised
Herpes simplex Virus-1	6	-
Varicella-Zoster Virus	1	-
*Haemophilus Influenzae*	2	-
*Streptococcus pneumoniae*	1	1
*Streptococcus pyogenes*	1	-
*Listeria monocytogenes*	-	1
*Mycobacterium tuberculosis*	4	4
*Sphingomonas paucimobillis*	1	-
*Burkholderia cepacia*	1	-
*Cryptococcus neoformans*	-	1
*Aspergillus*	1	-
*Haemophilus. influenzae and Cryptococcus neoformans*	1	-
Autoimmune encephalitis	4	-
SLE cerebritis	1	
Total	24	7

SLE = Systemic Lupus Erythematosus.

**Table 3:** Demographic features, clinical findings, CSF analysis, and radiological features for patients with encephalitis (*n* = 30; dual infection was excluded from this table).

		Total	Viral Cause	Bacterial Cause	Fungal Cause	Immune Cause	*p* Value
		*n* (%)	*n* (%)	*n* (%)	*n* (%)	*n* (%)	
A. Demographic profiles							
1. Age (*n* = 30)							0.088
	<35 years	13 (43.3)	2 (15.4)	6 (46.2)	0 (0)	5 (38.5)	
	35–59 years	11 (36.7)	3 (27.3)	6 (54.5)	2 (18.2)	0 (0)	
	≥60 years	6 (20.0)	2 (33.3)	4 (66.7)	0 (0)	0 (0)	
2. Sex (*n* = 30)							0.015
	Male	15 (50.0)	2 (13.3)	11 (73.3)	2 (13.3)	0 (0)	
	Female	15 (50.0)	5 (33.3)	5 (33.3)	0 (0)	5 (33.3)	
B. Clinical signs and symptoms							
Headache (*n* = 30)							0.553
	Present	17 (56.7)	4 (23.5)	9 (52.9)	2 (11.8)	2 (11.8)	
	Absent	13 (43.3)	3 (23.1)	7 (53.8)	0 (0)	3 (23.1)	
Seizure/convulsion (*n* = 29)							0.405
	Present	14 (48.3)	3 (21.4)	6 (42.9)	2 (14.3)	3 (21.4)	
	Absent	15 (51.7)	4 (26.7)	9 (60.0)	0 (0)	2 (13.3)	
Vomiting (*n* = 30)							0.164
	Present	9 (30.0)	2 (22.2)	4 (44.4)	2 (22.2)	1 (11.1)	
	Absent	21 (70.0)	5 (23.8)	12 (57.1)	0 (0)	4 (19.1)	
Changes in sensorium (*n* = 30)							0.161
	Present	25 (83.3)	4 (16.0)	15 (60.0)	2 (8.0)	4 (16.0)	
	Absent	5 (16.7)	3 (60.0)	1 (20.0)	0 (0)	1 (20.0)	
Sign of meningism (*n* = 27)							0.223
	Present	11 (40.7)	2 (18.2)	6 (54.5)	2 (18.2)	1 (9.1)	
	Absent	16 (59.3)	5 (31.3)	7 (43.7)	0 (0)	4 (25.0)	
Focal neurological deficit (*n* = 30)							0.362
	Present	7 (23.3)	1 (14.3)	5 (71.4)	1 (14.3)	0 (0)	
	Absent	23 (76.7)	6 (26.1)	11 (47.8)	1 (4.4)	5 (21.7)	
Constitutional symptoms (*n* = 29)							0.781
	Present	15 (51.7)	2 (13.3)	9 (60.0)	1 (6.7)	3 (20.0)	
	Absent	14 (48.3)	4 (28.6)	7 (50.0)	1 (7.1)	2 (14.3)	
Comorbidities (*n* = 30)							0.359
	Present	16 (53.3)	5 (31.1)	9 (56.3)	1 (6.3)	1 (6.3)	
	Absent	14 (46.7)	2 (14.3)	7 (50.0)	1 (7.1)	4 (28.6)	
GCS (*n* = 30)							0.729
	≤10	11 (36.7)	3 (27.3)	6 (54.5)	0 (0)	2 (18.2)	
	>10	19 (63.3)	4 (21.1)	10 (52.6)	2 (10.5)	3 (15.8)	
C. CSF analysis							
CSF protein (*n* = 28)							0.043
	Normal	11 (39.3)	4 (36.4)	2 (18.2)	1 (9.1)	4 (36.4)	
	Abnormal	17 (60.7)	3 (17.7)	12 (70.6)	1 (5.9)	1 (5.9)	
* CSF protein level (*n* = 28)		61 (5:246)	45 (19.3:72)	69.5 (5:246)	101.3 (37.6:165)	22 (21:68)	0.117
CSF pleocytosis (*n* = 28)							0.010
	Normal	12 (42.9)	4 (33.3)	3 (25.0)	0 (0)	5 (41.7)	
	Abnormal	16 (57.1)	3 (18.7)	11 (68.8)	2 (12.5)	0 (0)	
CSF: Blood Glucose Ratio (*n* = 26)							0.108
	Normal (≥0.5)	15 (57.7)	5 (33.3)	6 (40.0)	0 (0)	4 (26.7)	
	Abnormal (<0.5)	11 (42.3)	1 (9.1)	7 (63.6)	2 (18.2)	1 (9.1)	
* CSF glucose level (*n* = 28)		67 (18:136)	80 (61:136)	62 (18:109)	49.39 (32:66.78)	88 (57:100)	0.136
D. Radiological Investigations							
CT/MRI Results (*n* = 30)							0.118
	Normal	21 (70.0)	5 (23.8)	13 (61.9)	0 (0)	3 (14.3)	
	Abnormal	9 (30.0)	2 (22.2)	3 (33.3)	2 (22.2)	2 (22.2)	

* The data were shown with median (minimum, maximum). CT = Computed Tomography; MRI = Magnetic Resonance Imaging. *p* < 0.05 was the significant cutoff point.

**Table 4:** Univariate analysis of variables for predicting the survival of patients with encephalitis (*n* = 64).

		Total	Fatal Outcome	cOR	95% CI	*p* Value
		*n* (%)	*n* (%)			
Age						
	<35 years	25 (39.1)	2 (8.0)	Ref.		
	35–59 years	26 (40.6)	3 (11.5)	1.50	0.23, 9.83	0.673
	≥60 years	13 (20.3)	1 (7.7)	0.96	0.79, 11.67	0.973
Sex						
	Male	33 (51.6)	4 (12.1)	2.00	0.34, 11.78	0.444
	Female	31 (48.4)	2 (6.4)	Ref.		
Immune status						
	Immunocompetent	56 (87.5)	5 (8.9)	Ref.		
	Immunocompromised	8 (12.5)	1 (12.5)	1.46	0.15, 14.36	0.747
Seizure/convulsion						
	Present	26 (41.3)	4 (15.4)	3.18	0.54, 18.85	0.202
	Absent	37 (58.7)	2 (5.4)	Ref.		
Vomiting						
	Present	18 (28.1)	1 (5.6)	0.48	0.52, 4.44	0.520
	Absent	46 (71.9)	5 (83.3)	Ref.		
Changes in sensorium						
	Present	46 (73.0)	6 (100)	-	-	-
	Absent	17 (27.0)	0 (0.0)	-	-	-
Sign of meningism						
	Present	27 (45.0)	3 (11.1)	1.25	0.23, 6.76	0.796
	Absent	33 (55.0)	3 (9.1)	Ref.		
Focal neurological deficit						
	Present	12 (18.8)	2 (16.7)	2.40	0.39, 14.95	0.348
	Absent	52 (81.2)	4 (7.7)	Ref.		
Constitutional symptoms						
	Present	30 (51.7)	3 (10.0)	1.44	0.22, 9.36	0.700
	Absent	28 (48.3)	2 (7.1)	Ref.		
Comorbidities						
	Present	31 (48.4)	2 (6.4)	0.50	0.08, 2.95	0.444
	Absent	33 (51.6)	4 (12.1)	Ref.		
GCS						
	≤10	19 (29.7)	3 (15.8)	2.63	0.48, 14.38	0.266
	>10	45 (70.3)	3 (6.7)	Ref.		
CSF protein						
	Normal	30 (49.2)	4 (13.3)	Ref.		
	Abnormal	31 (50.8)	2 (6.4)	0.45	0.08, 2.65	0.376
CSF pleocytosis						
	Normal	28 (45.9)	1 (3.6)	Ref.		
	Abnormal	33 (54.1)	5 (15.1)	4.82	0.53, 44.00	0.163
CSF: Blood Glucose Ratio						
	Normal	37 (63.8)	3 (8.1)	Ref.		
	Abnormal	21 (36.2)	3 (14.3)	1.89	0.35, 10.33	0.463
CT/MRI Results						
	Normal	43 (70.5)	4 (9.3)	Ref.		
	Abnormal	18 (29.5)	2 (11.1)	1.22	0.20, 7.33	0.829

CT = Computed Tomography; MRI = Magnetic Resonance Imaging. *p* < 0.05 was the significant cutoff point.

## Data Availability

The datasets generated and/or analyzed during the current study are available in the manuscript.
